# Injection of α-syn-98 Aggregates Into the Brain Triggers α-Synuclein Pathology and an Inflammatory Response

**DOI:** 10.3389/fnmol.2019.00189

**Published:** 2019-08-06

**Authors:** Tingfu Du, Zhengcun Wu, Haiyu Luo, Shuaiyao Lu, Kaili Ma

**Affiliations:** ^1^Center for Drug Safety Evaluation and Research, Institute of Medical Biology, Chinese Academy of Medical Sciences, Kunming, China; ^2^Medical Primate Research Center & Neuroscience Center, Chinese Academy of Medical Sciences, Beijing, China; ^3^Yunnan Key Laboratory of Vaccine Research Development on Severe Infectious Diseases, Kunming, China

**Keywords:** α-syn-98 aggregates, Parkinson’s disease, α-syn pathology, gliosis, inflammatory response

## Abstract

Pathological aggregation of α-synuclein (α-syn) is a major component of Lewy bodies (LB), which play a central role in pathogenesis of Parkinson’s disease (PD). Differential expression of α-syn isoforms has been shown in PD. Isoform α-syn-98 is generated by excision of exon-3 and exon-5 of the α-syn gene. In contrast to the canonical full-length α-syn isoform (α-syn140), little is known about the function of the α-syn-98 isoform. In the present study, to identify the potential role of α-syn-98 protein in PD, we examined the effects of exogenous recombinant insoluble α-syn-98 aggregates on α-syn pathology and inflammatory responses in the midbrain. After injection of α-syn-98 aggregates into the substantia nigra (SN), mice exhibited motor dysfunction accompanied by nigral dopaminergic neuron loss. In addition, α-syn-98 aggregates injection resulted in a significant increase in phosphorylation of endogenous α-syn. Accumulations of α-syn were co-localized with p62 and ubiquitin, which suggests α-syn-98 aggregates-induced pathology exhibits properties similar to human LB. Many glial cells were activated after α-syn-98 aggregates injection. In addition, expression of NF-κB, interleukin 6 (IL6), and tumor necrosis factor-α (TNF-α) and levels of oxidative stress increased after α-syn-98 aggregates injection. Our results suggest that α-syn-98 may play a crucial role in the pathogenesis of PD.

## Introduction

Parkinson’s disease (PD) is one of the most common age-related neurodegenerative disorders and is typified by progressive loss of dopaminergic neurons in the substantia nigra pars compacta (SNc; Goedert et al., 2017; Sun et al., 2018). The classical pathological hallmark of PD is Lewy body (LB) inclusions, and the major component of LB inclusions is α-synuclein (α-syn; Kim et al., 2014). In the physiological condition, α-syn is a kind of soluble protein and performs multiple physiological functions in the nervous system, such as neurotransmitter release, synaptic function and plasticity. It is enriched in the central nervous system (Bartels et al., 2011), but accumulations of α-syn in neurons are toxic. Evidence suggests that misfolded α-syn plays an important role in the pathogenesis of PD (Wong and Krainc, 2017).

A number of post-translational modifications of α-syn have been found in both familial and sporadic PD (Oueslati et al., 2010). In addition to several post-translational modifications, at least four alternatively spliced α-syn mRNA transcripts have been identified in humans (McLean et al., 2012; Beyer and Ariza, 2013). In addition to the full-length isoform, α-syn140 (contains 140 amino acids), there are three alternative variants, α-syn126, α-syn112, and α-syn-98 (Beyer et al., 2008a,b; Brudek et al., 2016). α-syn-98 (contains 98 amino acids) is generated by in-frame excision of exon 3 and exon 5 (Beyer et al., 2008a,b). Previous studies have shown that expression of α-syn-98 is elevated in PD, and loss of exon 5 might increase α-syn aggregation (Bungeroth et al., 2014). Although alterations in α-syn isoforms have been associated with aggregation in PD, the biological and pathological significance of α-syn-98 remains unknown.

Emerging evidence indicates a strong correlation between α-syn aggregation and neuroinflammation dysfunction. Neuroinflammation results primarily from activated glial cells (astrocytes and microglia) in the brain, and an increased number of glial cells has been reported in PD (Pal et al., 2016). It has been suggested that glial reaction and inflammatory processes participate in the pathology of PD (Niranjan, 2014). It was demonstrated that injection of α-syn into the SN is sufficient to affect dopaminergic neurotransmission and leads to a loss of neurons (Wawer et al., 2016). However, until now, little was known about the effects of α-syn-98 on the initiation of neuroinflammation and neurodegeneration. In our previous study, we have found that the over-expression of α-syn-98 in dopaminergic cells (*in vitro*) promoted the aggregate formation and significantly decreased cell viability when treated with rotenone (Ma et al., 2011). It has been reported α-syn aggregates were emerging as crucial factors in the pathogenesis of synucleinopathies (Soto and Pritzkow, 2018; Wang et al., 2019). In the present study, we investigated the influence of intracerebral administration of exogenous α-syn-98 aggregates on α-syn pathology and inflammatory responses in the mouse brain.

## Materials and Methods

### Plasmid Construction and Recombinant α-syn-98 Purification

Human α-syn-98 cDNA was amplified from the template pDNA3.1-α-syn-98, which we constructed in our previous study (Ma et al., 2011), using the forward primer 5′-TTCCCGGGTATGGATGTATTCATG-3′ and the reverse primer 5′-AAGCGGCCGCTTAGGCTTCAGGTTCG-3′. For bacterial expression of the GST-α-syn-98 fusion protein, the cDNA was subcloned into the *Sma*I and *Not*I restriction sites of the pGEX-5X-1 vector (ampicillin resistant, GE Healthcare Life Sciences), which encodes a 26-kD N-terminal GST tag that can be removed *via* site-specific proteolysis using highly pure Factor Xa protease (NEB, America). Accuracy and orientation of the expression constructs were identified by restriction digestion and nucleotide sequencing analyses.

The GST-α-syn-98 fusion construct (pGEX-5X-1-α-syn-98) was transformed into the *E. coli* BL21 strain for protein expression. Transformed bacteria were grown in Luria-Bertani medium containing 100 μg/mL ampicillin at 37°C until the OD_600_ was approximately 0.8. Next, IPTG was added to a final concentration of 1.0 mM, and the culture was incubated for an additional 6 h. The cell pellet from 100 mL of induced culture was collected by centrifugation and resuspended in ice-cold PBS (pH 7.4). GST-α-syn-98 was purified by BeaverBeads^TM^ GST-tag protein purification beads (BEAVER, America) according to the manufacturer’s instructions. Purity was analyzed by SDS-PAGE, and concentration was measured using a bicinchoninic acid (BCA) protein assay kit (BWBIO, China). The obtained protein was concentrated using a 10-kD Millipore filter at 4,000× *g* for 30 min at 4°C and then diluted into an appropriate amount of enzyme buffer (50 mM Tris-HCl, pH 8.0, 100 mM NaCl, 5 mM CaCl_2_). An appropriate quantity of factor Xa protease was added according to the manufacturer’s instructions. Following an overnight incubation at 16°C, the buffer was exchanged to buffer A using a 3-kD Millipore filter. The GST tag was then removed by co-incubating the mixture with the GST beads as described above. The identity of recombinant α-syn-98 was verified by western blot analysis.

### Recombinant α-syn-98 Aggregates Preparation and Identification

Purified recombinant α-syn-98 (8.6 mg/mL, added 0.1% NaN_3_) was incubated at 37°C on a shaking table at 1,000 rpm for 6 days. Next, α-syn-98 aggregates were centrifuged at 12,000 rpm for 20 min (NaN_3_ in the supernatant were removed) and then resuspended in PBS. The aggregates formation was monitored by Thioflavin T (ThT) assay for up to 8 days using 450 nm excitation and emission intensity at 500 nm by Multifunctional Enzyme Reader (Varioskan LUX, Thermo). The aggregates were incubated with ThT at the final concentration of 25 μM for 30 min at room temperature before reading on an Ultra Evolution 384 plate reader (Corning, NY, USA). The presence of amyloid-like fibrils typically results in readings that are 20–100-fold higher than the same concentration of monomeric protein (Polinski et al., 2018). Recombinant α-syn-98 aggregates and monomer were also treated with 5 μg/ml proteinase K for 30 min at 37°C to evaluate its proteinase K resistance as previously described, but for several improvements (Miake et al., 2002; Cremades et al., 2012). The digests were analyzed by SDS-PAGE. Then, 10 μL of aggregates was spotted on carbon-coated EM grids and incubated for 5 min at room temperature. The grids were washed with 100 μL Millipore-filtered water and then negatively stained with 1% uranyl acetate for 5 min. Afterward, the grids were washed with 100 μL Millipore-filtered water and dried with compressed air. EM images were obtained with a Hitachi TEM system at an accelerating voltage of 100 kV.

### Animals

C57BL/6J mice at 9–10 weeks of age (18–25 g; Vital River Animal Technology Co., Limited, Beijing, China) were housed in groups of two animals per cage under a 12-h light/dark cycle at constant temperature (25°C) with *ad libitum* access to food and water. All C57BL/6J mice were allowed 2 weeks of habituation to the housing conditions before the start of experiments. This study was carried out in accordance with the recommendations of the Yunnan Province Experimental Animal Management Association and the Experimental Animal Ethic Committee of the Institute of Medical Biology Chinese Academy of Medical Sciences. The protocol was approved by the Experimental Animal Ethic Committee of the Institute of Medical Biology Chinese Academy of Medical Sciences.

### Stereotaxic Surgery

Prior to surgery, 11- to 12-week-old C57BL/6J mice were anesthetized with 20 mg/kg pentobarbital sodium. The α-syn-98 group (22 males and 22 females) was injected bilaterally with 8 μg (4 μg/side, 2 μL) of insoluble recombinant α-syn-98 aggregates (anterior-posterior: −3.1 mm; medial-lateral: ±1.3 mm; dorsal-ventral: −4.5 mm from Bregma and the dura) using a 10-μL Hamilton syringe. The control group (22 males and 22 females) was bilaterally injected with 2 μL sterile PBS.

Some studies revealed that injection of α-synuclein fibrils into mouse brains induced α-synuclein pathology at 1 month after injection (Masuda-Suzukake et al., 2014; Luk et al., 2016), so we also selected the time point at 1 month for further study.

### Rotarod Test

The rotarod test was used to estimate the balance and motor coordination of mice, which used an accelerating rotarod (Shanghai, YLS-4C). Briefly, the mice were placed on 3 cm-diameter rods and measured the time each animal was able to maintain its balance. Animals received 2 days of training before the start of experiments. The speed of the rotarod accelerated from 0 to 40 rpm in 2 min and was maintained at 40 rpm for 3 min. The latency to fall off the rod during this period was recorded. Trials were performed in triplicate. The mean of latency to fall off the rod was used for analysis.

### Pole Descent Test

A pole, which was 0.5 m long and 1 cm in diameter, was wrapped with non-adhesive shelf liner to facilitate grip and placed in the home cage. Animals received 2 days of training to descend from the top of the pole into the home cage. On the test day, animals were placed head-down on the top of the pole, and the time to descend back into the home cage was recorded. Timing began when the experimenter released the animal and ended when one hind limb reached the home cage. Trials were performed in triplicate.

### Immunohistochemistry (IHC) and Immunofluorescence

Mice were deeply anesthetized by a pentobarbital injection (20 mg/kg) and perfused with 0.1 M phosphate buffer followed by 4% paraformaldehyde solution. The brains were fixed in a 4% paraformaldehyde solution overnight and then embedded in paraffin. The paraffin-embedded tissues were sectioned at 4 μm using a microtome (Leica, RM2235, Germany). After antigen retrieval with sodium citrate buffer in a microwave oven and a 15-min incubation with 3% H_2_O_2_ to block endogenous peroxidase activity, sections on slides were incubated with appropriate antibodies overnight at 4°C for immunohistochemistry (IHC). Antibodies used in this study are summarized in Table 1. After washing three times with PBS, the sections were incubated with biotin-conjugated secondary antibodies (SP Rabbit and Mouse HRP Kit, CW2069) for 1 h at room temperature and visualized using 3,3-diaminobenzidine (DAB).

**Table 1:** Antibodies used in this study.

Primary antibodies	Type	Source	WB	IF/IHC
Anti-α-synuclein	Mouse mono	ENZO (ALX-804-656)	1/1,000	-
Anti-α-synuclein	Mouse poly	BD (610787)	1/2,000	-
Anti-α-synuclein (phospho S129)	Mouse mono	Abcam (ab184674)	-	1/500
Anti-SQSTM1/p62	Rabbit poly	Abcam (ab91526)	-	1/200
Anti-ubiquitin antibody	Rabbit poly	Abcam (ab7780)	-	1/100
Anti-GFAP	Mouse mono	Sigma (G3893)	1/1,000	1/1,000
Anti-GFAP	Rabbit poly	Abcam (ab7260)	1/1,000	1/1,000
Anti-Iba1	Rabbit poly	Wako (016-20001)	-	1/200
Anti-Iba1	Goat poly	Abcam (ab5076)	1/1,000	-
Anti-tyrosine hydroxylase	Rabbit poly	Abcam (ab117112)	1/3,000	1/1,000
Anti-GAPDH	Rabbit mono	Abcam (ab181602)	1/1,000	
Anti-mouse IgG H&L (Alexa Fluor^®^ 488)	Goat poly	Abcam (ab6671)	-	1/200
Anti-rabbit IgG H&L (Alexa Fluor^®^ 594)	Goat poly	Abcam (ab150080)	-	1/200

For immunofluorescence staining, the step of blocking endogenous peroxidase activity was omitted. After incubating with primary antibodies at 4°C overnight, the slides were washed three times with phosphate buffer and incubated with Alexa Fluor-conjugated secondary antibodies (Table 1) for 1 h at room temperature. They were then washed three times with phosphate buffer and coverslipped in fluoroshield mounting medium with DAPI (Abcam, ab104139).

Images were captured using a Nikon microscope (Nikon Eclipse 80i) with NIS-Elements software and analyzed using ImageJ software (NIH, Bethesda, MD, USA). At least three brain sections from each subject were chosen for analysis (*n* = 4–6 per treatment).

Estimation of the number of tyrosine hydroxylase (TH)-positive neurons in the SN was based on the method of Smeyne and colleagues (Hamre et al., 1999; Baquet et al., 2009). Mouse brains were serially sectioned from the rostral hippocampus to the anterior aspects of the cerebellar-midbrain junction. We performed our analysis by sampling sections equally spaced every 100 μm, once sections in each brain were counted, we summed the cell totals, multiplied by 2 to correct for uncounted sections. In this study, at least 10 slides from rostro to caudal SN were selected to estimate the numbers of TH-neurons. Each side of the brain for each animal was counted and then generated a total dopaminergic SN neuron count.

### Western Blot

Sixteen mice (eight α-syn-98 and eight controls) were sacrificed, midbrain was rapidly extracted (between interbrain and hindbrain) which referred to mouse brain map^1^ and collected in tubes with protein lysis buffer supplemented with 2 mM PMSF and protease inhibitor cocktail (Merck, 539131). Samples were then homogenized until there were no visible tissue pieces. The homogenate was transferred to a new tube and centrifuged at 20,000× *g* for 15 min at 4°C. Protein concentrations were measured using a BCA method (BCA Protein Assay Kit, CW0014S, CWBIO). Protein samples were separated on Criterion TGX Stain-Free gels (Bio-Rad) for 120 min at 85 V. The gel was then transferred to a polyvinylidene fluoride (PVDF) membrane (Millipore, Bedford, MA, USA) in 5 min using the Trans-Blot Turbo Transfer System (Bio-Rad). After transfer, the membrane was blocked with 5% nonfat milk buffer for 1 h at room temperature with gentle agitation. The membrane was then incubated with primary antibodies overnight at 4°C. The antibodies used in this study are listed in Table 1. After incubation with primary antibodies, the blotting membrane was washed 3 × 15 min with TBST. Then, membranes were incubated with the following secondary antibodies for 1 h at room temperature: anti-mouse IgG H&L-HRP (1:5,000, KPL, 5220-0341), anti-rabbit IgG H&L-HRP (1:5,000, KPL, 5220-0336), or anti-goat IgG H&L-HRP (1:5,000, Abcam, ab6885) for 1 h at room temperature. Next, each blotting membrane was washed 3 × 15 min with TBST. The enhanced chemiluminescence (ECL) method was used to detect signals. Band intensities were imaged using the ChemiDoc MP imaging system (Bio-Rad) and quantified using ImageLab software (version 4.1, Bio-Rad).

Soluble and insoluble α-syn isolation was performed as previously described (Harms et al., 2013; Ordonez et al., 2018) with little changes. Briefly, each sample of midbrains was homogenized in 500 μL of lysis buffer (50 mM Tris, pH 7.4, 150 mM NaCl, 5 mM EDTA) containing protease inhibitor cocktail (Millipore, America). And Triton X-100 was added to homogenates at a final concentration of 1%. Following 30 min incubation on ice, the homogenate from each sample was centrifuged (20,000× *g*) at 4°C for 1 h. Supernatants from each sample were transferred to a fresh tube and designated as “soluble α-syn.” Pellets were solubilized in lysis buffer containing 2% SDS and, following incubating on ice at 30 min, were designated as “insoluble α-syn.” Soluble and insoluble α-syn was subsequently immunoblotted as described above.

### Quantitative Real-Time PCR (qPCR) and ELISA

NF-κB, tumor necrosis factor-α (TNF-α), and interleukin-6 (IL-6) mRNA levels were measured in total RNA samples from different midbrain of eight mice (four α-syn-98 and four controls) utilizing SYBR Green-based Quantitative real-time PCR (qPCR) performed in 96-well plates on a CFX96 Real-Time PCR Detection System (Bio-Rad). GAPDH was used as a reference gene. Briefly, 1 μg of total RNA from each sample was reverse transcribed to cDNA [Eastep^®^ RT Master Mix (5X) Kits, LS2054, Promega, USA]. qPCR was performed using Eastep^®^ qPCR Master Mix (2X; LS2068, Promega) according to the manufacturer’s recommendations. All genes were analyzed in triplicate. Primers were as follows: *gapdh* 5′-TGTGTCCGTCGTGGATCTGA-3′ and 5′-TTGCTGTTGAAGTCGCAGGAG-3′, *nf-κb* 5′-TGCGATTCCGCTATAAATGCG-3′ and 5′-ACAAGTTCATGTGGATGAGGC-3′, *il-6* 5′-CTGCAAGAGACTTCCATCCAG-3′ and 5′-AGTGGTATAGACAGGTCTGTTGG-3′, *tnf-α* 5′-CTGAACTTCGGGGTGATCGG-3′ and 5′-GGCTTGTCACTCGAATTTTGAGA-3′. The comparative Ct (2^ΔΔCt^) method was used to quantify expression of genes, and fold change (FC) was used to present data.

NF-κB, IL-6, and TNF-α ELISA assays were performed using murine NF-κB, IL-6, and TNF-α ELISA Kits (Bossbio, China) according to the manufacturer’s protocol (eight α-syn-98 and eight controls). Plates were read on a TECAN instrument (TECAN, Infinite^®^M20). Briefly, ELISA plates were loaded with HRP-labeled capture antibodies. After samples or standards were added to the wells, antigen-enzyme-antibody complexes were formed. Next, plates were washed, and TMB substrate solution was added. HRP catalyzes the conversion of TMB into a blue product. The reaction was terminated by the addition of a sulfuric acid solution, and color intensity was measured spectrophotometrically at a wavelength of 450 nm. Protein concentration in samples was determined by comparing the optical density of each sample to a standard curve.

### Levels of Superoxide Dismutase (SOD), Malondialdehyde (MDA), and Glutathione (GSH)

Since oxidative stress is believed to be an important contributor to the neurodegenerative process in PD (De Lazzari et al., 2018), we analyzed superoxide dismutase (SOD), MAD, and glutathione (GSH) levels in another eight brains injected with α-syn-98 aggregates. Mouse midbrain tissues were homogenized on ice and processed using total SOD activity detection kit (WST-8 method), Malondialdehyde (MDA) detection kit and GSH ELISA kit respectively, according to the manufacturer’s instructions (Beyotime, China).

### Statistics and Image Processing

Statistical analyses were performed using GraphPad Prism software (GraphPad). Differences between groups were analyzed using the Student’s *t*-test. Images were processed using Adobe Photoshop CS6.

## Results

### Purification of Insoluble α-syn-98 Aggregates

**Figure 1:**
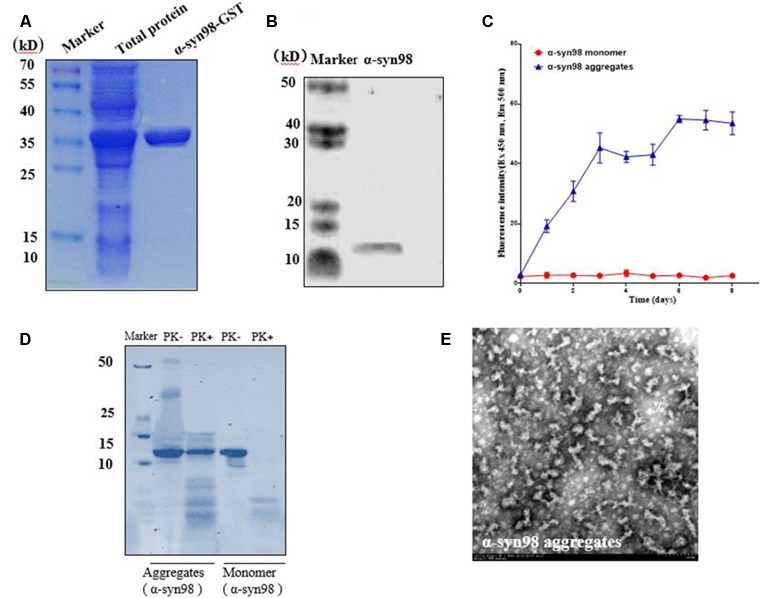
Preparation of recombinant α-syn-98 aggregates. **(A)** Quality of purified recombinant fusion protein α-syn-98-GST was estimated by SDS-PAGE, and gels were stained with Coomassie brilliant blue. Lane 1: total protein obtained from *E. coli* BL21 expressing recombinant plasmid (pGEX-5X-1-α-syn-98). Lane 2: α-syn-98-GST protein purified from lysates using GST fusion protein purification beads (BeaverBeads^TM^ GSH). **(B)** Western blot analysis of purified α-syn-98. Mouse monoclonal antibody specific for α-syn (ALX-804-656, ENZO) was used. **(C)** The aggregates formation monitored by ThT assay for up to 8 days. **(D)** The proteinase K resistance (5 μg/ml) of recombinant α-syn-98 aggregates analysis by SDS-PAGE, and gels were stained with Coomassie brilliant blue. **(E)** EM determined the conformation of α-syn-98 aggregates obtained by incubation for 6 days at 37°C.

Recombinant plasmid (pGEX-5X-1-α-syn-98) was expressed in *E. coli* BL21 and analyzed by SDS-PAGE. Overexpressed recombinant fusion protein α-syn-98-GST had an apparent molecular mass of 36 kD, which was consistent with the predicted size (Figure 1A). As showed in Figure 1B, α-syn-98 protein without the GST tag was about ~11 kD by western blot analysis (Figure 1B). When incubated at 37°C on a shaking table at 1,000 rpm, the formation of aggregates was monitored by ThT assay for up to 8 days. The detected fluorescence value of α-syn-98 aggregates was 20-fold higher than that of monomers at the 6th day and tended to be stable in the following 2 days (Figure 1C), proving that α-syn-98 could form stable aggregates after 6 days of culture. Therefore, we used aggregates cultured for 6 days to carry out follow-up experiments. α-syn fibrils has been reported to increase the resistance of proteinase K degradation both *in vitro* and *in vivo*. While the monomeric form of the protein is highly susceptible to degradation (Neumann et al., 2002). We found that the almost of aggregates of α-syn-98 were resistant to degradation when treated with 5 μg/ml proteinase K for 30 min at 37°C but monomer was degraded completely (Figure 1D). EM showed that α-syn-98 protein formed short rod aggregates (Figure 1E), which was clearly distinct from α-syn140 as reported by Bungeroth et al. (2014).

### α-syn-98 Injected Mice Displayed Motor Dysfunction in the Rotarod and Pole Descent Tests

**Figure 2:**
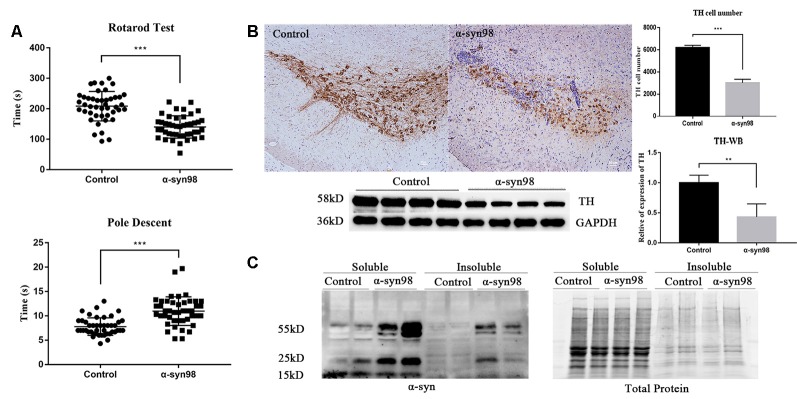
C57 mice injected with α-syn-98 aggregates performed similarly to other Parkinson’s disease (PD) model mice. **(A)** Mice injected with α-syn-98 aggregates showed a decrease in time during the rotarod test and an increase in time during the pole descent test at 1 month after injection; Control (*n* = 44); α-syn-98 aggregates (*n* = 44). Error bars represent SD. ***p < 0.001. **(B)** Injection of α-syn-98 aggregates into the substantia nigra (SN) of C57 mice led to a significant decrease in tyrosine hydroxylase (TH) in the midbrain. Immunohistochemistry (IHC) staining for TH in the midbrain (top). Scale bar, 50 μm. Counts of TH-positive neurons. Control (*n* = 4); α-syn-98 aggregates (*n* = 4). Western blotting with a TH antibody is shown (bottom). Control (*n* = 8); α-syn-98 aggregates (*n* = 8). Error bars represent SD. **p < 0.01. **(C)** α-syn-98 aggregates resulted in the accumulation of higher molecular weight α-syn species in soluble and insoluble fractions of midbrain homogenate. α-syn mouse polyclonal antibody (BD, 610787) specific for α-syn was used. Control (*n* = 2); α-syn-98 aggregates (*n* = 2).

To examine if α-syn-98 aggregates injection results in behavioral deficit in mice, we assessed motor behavior by rotarod test and pole test at 1 month. Balance and motor coordination were assessed using the accelerated rotarod task. Bradykinesia was assessed using pole test. The rotarod test revealed that α-syn-98 aggregates injection mice stayed on the rotating rod for a significantly shorter duration than the control group (Figure 2A, *P* < 0.0001). The same trend was observed in the pole test, with it found that the α-syn-98 aggregates group took a significantly longer time to descend the pole (Figure 2A, *P* < 0.0005).

### PD-Like Pathologies in C57 Mice Are Evident After α-syn-98 Aggregates Injection

In addition, PD-like pathologies in mice were evident within 1 month after α-syn-98 aggregates injection. Mice injected with α-syn-98 aggregates led to a significant decrease in TH in the midbrain (Figure 2B). Finally, injection of aggregates resulted in the accumulation of higher molecular weight α-syn species, both in soluble and insoluble fractions of midbrain homogenate (Figure 2C).

### Aggregates Were Co-localized With p62 and Ubiquitin

**Figure 3:**
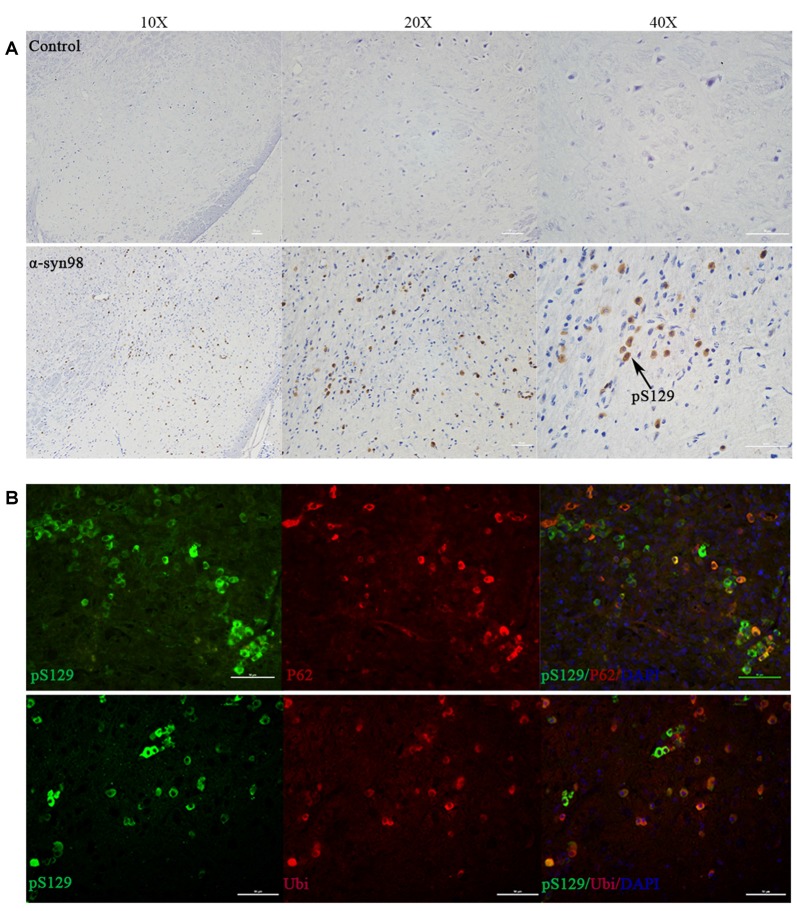
Aggregates were co-localized with p62 and ubiquitin. **(A)** pS129 inclusions observed in the midbrain of C57 mice after injection of α-syn-98 aggregates (IHC). Scale bar, 50 μm. **(B)** Double staining with P62, ubiquitin (Ubi) and pS129. Scale bar, 50 μm. Control (*n* = 6); α-syn-98 aggregates (*n* = 6).

We confirmed α-syn inclusion formation in the mouse midbrain with IHC using anti-phospho-Ser129 of α-syn (pS129) antibody (Figure 3A). We observed pS129 accumulation in the midbrain after injection of α-syn-98 and complete absence of pS129 aggregates in animals injected with an equal volume of PBS. Accumulation of pS129 inclusions did not spread widely in the brain depending on the region examined. Most α-syn pathologies was restricted in the midbrain. Most of the pS129 inclusions were also positive for antibodies to p62/SQSTM1 and ubiquitin (Kuusisto et al., 2003). It is well known that p62, an adaptor protein, is a component of LB and neurofibrillary tangles. Therefore, we examined whether p62 co-localized with α-syn inclusions were generated in the brains. The results showed that p62 apparently accumulated at pS129-positive inclusions (Figure 3B). Ubiquitin also colocalized with pS129 (Figure 3B). Our findings showed that α-syn inclusions were phosphorylation-, p62-, and ubiquitin-positive consistent with features of PD.

### Injection of α-syn-98 Aggregates Induces Gliosis in the Midbrain

Emerging evidence indicates a strong correlation between α-syn aggregation and neuroinflammation dysfunction. Neuroinflammation in the brain results primarily from activated glial cells (astrocytes and microglia) in the brain, and an increased number of glial cells has been reported in PD. To explore the effect of α-syn-98 aggregates on glial cells, we conducted IHC and western blot analysis to assess astrocytes and microglia in the brain. We observed widespread astrocytosis and microgliosis after α-syn-98 aggregates injection into the midbrain.

**Figure 4:**
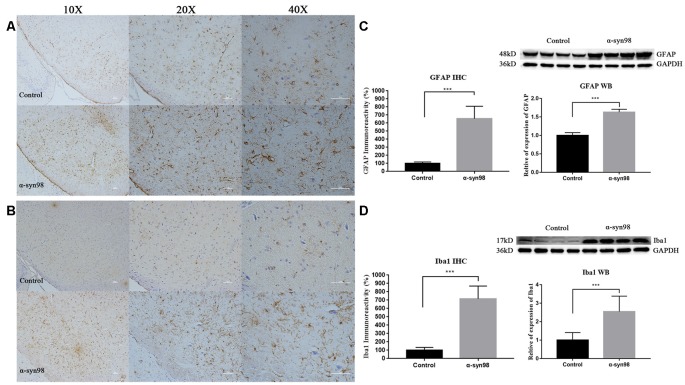
Injection of α-syn-98 aggregates induced gliosis in the midbrain. **(A)** IHC staining for glial fibrillary acidic protein (GFAP) in the midbrain. **(B)** Western blotting with a GFAP antibody is shown. **(C)** IHC staining for Iba1 in the midbrain. **(D)** Western blotting with an Iba1 antibody is shown. Scale bars: 50 μm. IHC, Control (*n* = 6); α-syn-98 aggregates (*n* = 6). Western blotting, Control (*n* = 8); α-syn-98 aggregates (*n* = 8). Error bars indicate SD. ***p < 0.001.

Quantification of IHC and western blots showed that glial fibrillary acidic protein (GFAP) and Iba1 increased significantly in the midbrain when compared with the control group (Figures 4A–D). These results clearly indicate that exogenous α-syn-98 aggregates activate astrocytes and microglia.

### NF-κB, IL-6, TNF-α and Levels of Oxidative Stress Increase Significantly in the Midbrain After Injection of α-syn-98 Aggregates Into the SN

**Figure 5:**
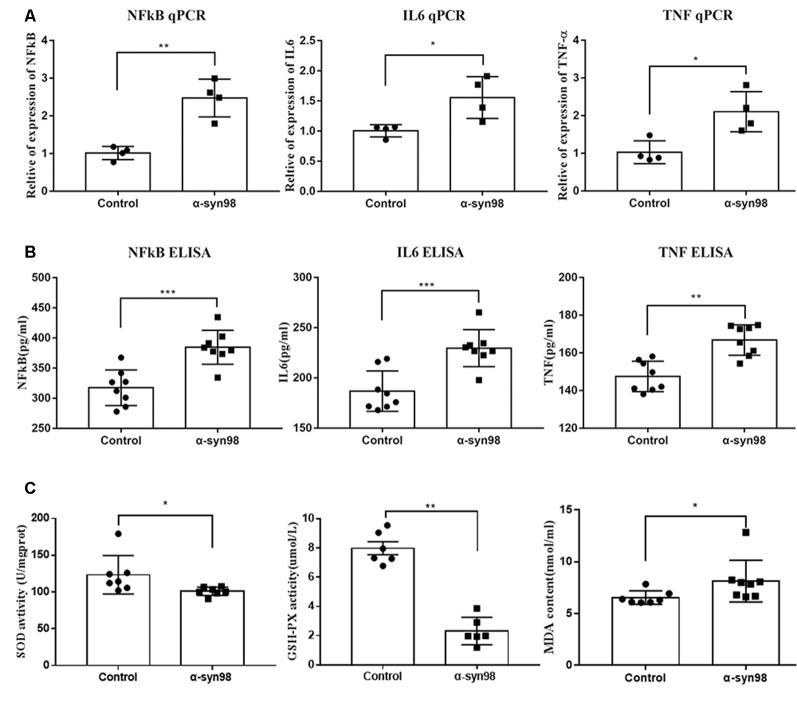
NF-κB, interleukin-6 (IL-6), tumor necrosis factor-α (TNF-α) and malondialdehyde (MDA), glutathione (GSH), and superoxide dismutase (SOD) increased significantly in the midbrain after injection of α-syn-98 aggregates into the SN. **(A)** Expression of NF-κB, IL-6, and TNF-α mRNA in the midbrain by Quantitative real-time PCR (qPCR). **(B)** Expression of NF-κB, IL-6, and TNF-α protein in the midbrain by ELISA. qPCR, Control (*n* = 4); α-syn-98 aggregates (*n* = 4). ELISA, Control (*n* = 8); α-syn-98 aggregates (*n* = 8). Error bars indicate SD. *p < 0.05, **p < 0.01, ***p < 0.001. **(C)** Concentrations of SOD were analyzed at a wavelength of 450 nm. Concentrations of GSH were measured at a wavelength of 405 nm using an ELISA kit. Concentrations of MDA were measured at a wavelength of 532 nm. Control (*n* = 6–8), α-syn-98 aggregates (*n* = 6–8), Error bars indicate SD. *p < 0.05, **p < 0.01.

We used ELISA and qPCR to evaluate the expression of NF-κB/p65, IL-6, and TNF-α in the midbrains of mice following injection of α-syn-98 into the SN. After α-syn-98 aggregates injection into the SN, we observed significant increases in levels of NF-κB/p65, IL-6, and TNF-α in the midbrain when compared with the control group (Figure 5). Oxidative stress is a prevalent factor in the pathogenesis of PD. The relationship between α-syn aggregation and oxidative stress is likely to be a vicious cycle. SOD can protect cells from toxicity of free radical damage. As the main free radical scavenger, SOD levels reflect the ability of cells to protect themselves against oxidative stress reactions. GSH is a small molecule peptide composed of three amino acids and is an important antioxidant *in vivo*. The amount of GSH is an important factor in measuring the antioxidant capacity of the body. MDA is a product of lipid peroxidation, and its content indirectly reflects the metabolic generation of free radicals. Therefore, to assess oxidative stress levels, we used enzymatic methods to measure SOD, MDA, and GSH concentrations in the midbrains of C57 mice. As showed in Figure 5, our results indicated decreases in SOD and GSH levels and an increase in MDA levels when compared with control mice. As key substances in oxidative stress processes, changes in levels of SOD, GSH, and MDA suggest that α-syn-98 aggregates may promote oxidative stress in the mouse brain.

## Discussion

Alpha-synuclein plays a number of indispensable functions under normal physiological conditions; however, pathological aggregation of α-syn may play a role in the occurrence of PD (Lashuel et al., 2013). It has been shown that α-syn isoform expression is differentially regulated in the brains of patients with PD and other LB disorders (Beyer et al., 2008a,b; Brudek et al., 2016). Truncated forms of α-syn have been shown to enhance the formation of aggregates that may be involved in LB formation (Bungeroth et al., 2014). α-syn-98, which contains 98 amino acids, is characterized by deletion of exons 3 and 5 (McLean et al., 2012). As the shortest isoform of α-syn, α-syn-98 is missing 14 N-terminal amino acids and 28 C-terminal amino acids. As a result, α-syn-98 is thought to be missing most functional domains, including sites of post-translational modification and mutation in exons 3 and 5. In a study of aggregation properties of α-syn-98, it was shown that α-syn-98 forms annular structures when compared with the well-known straight fibrils of α-syn140 (Bungeroth et al., 2014). α-syn-98 may be the most amyloidogenic and toxic of the alternatively spliced forms of α-syn due to the preservation of critical domains that include aggregation-prone sequences (Beyer et al., 2009). Overexpressed α-syn-98 has shown a significant tendency for the formation of aggregate and cytotoxicity when exposed to rotenone (Ma et al., 2011). Alpha-synuclein aggregation is one central hallmark of neurodegeneration in PD (Prots et al., 2018). In this study, it is possible that exploring roles of the α-syn-98 aggregates in the brains may deepen our understanding of the pathology of PD.

PD is characterized neuropathologically by intracellular aggregates of fibrillary α-syn. In addition, α-syn is phosphorylated at Ser129 and partially ubiquitinated in PD (Hasegawa et al., 2002). Most α-syn deposited as LB is phosphorylated at Ser129 in PD brains (Arawaka et al., 2017). Native α-syn is unfolded, but it readily assembles into amyloid-like aggregates under appropriate conditions (Yonetani et al., 2009; Ghosh et al., 2013). It has been demonstrated that intracerebral injection of α-syn fibrils or insoluble α-syn converts α-syn into pathological form (Luk et al., 2012; Masuda-Suzukake et al., 2013). In this study, we tested whether insoluble α-syn-98 aggregates could induce PD-like α-syn pathologies in mice. After 4 weeks of injections, we observed abundant phospho-α-syn pathologies in the midbrain. It indicated that α-syn-98 aggregates efficiently seed aggregation and fibrillization of soluble endogenous α-syn in the midbrain since α-syn-98 lack of Ser129. Ubiquitin-binding protein p62, which is encoded by the SQSTM1 gene, is a widely expressed cytoplasmic protein that can bind to ubiquitin and is involved in several signaling pathways. The interaction of p62 and ubiquitin (Ubi) contributes to the formation of α-syn inclusions. Progressive accumulation of α-syn inclusions is usually accompanied by p62 and ubiquitin in PD (Kuusisto et al., 2001). In our study, p62 and ubiquitin apparently accumulated at phospho-α-syn-positive inclusions, which is consistent with pathological features of LB and Lewy neuritis (Kuusisto et al., 2001; Watanabe et al., 2012). The results of the present study clearly demonstrate that α-syn-98 aggregates trigger PD-like α-syn pathologies and induce motor dysfunction with degeneration of dopaminergic neurons.

Onset and progression of PD are accompanied by an immune response and inflammation. Neuroinflammation in the brain results primarily from chronically activated glial cells (astrocytes and microglia) and is a common feature in the pathology of PD (Niranjan, 2014). Neuroinflammation is an acutely protective mechanism. However, long-lasting and persistent formation and accumulation of pro-inflammatory mediators can initiate neuronal damage and neurodegeneration (Glass et al., 2010). Activation of astrocytes has been generally described in the SN of patients with PD (Solano et al., 2008). Astrocytes recognize various signals from activated microglia, such as soluble chemokines and cytokines, and function differentially (Mena and Garcia, 2008). The role of astrocytes is not fully understood and still under debate. Neuroprotective and neurodegenerative functions of astrocytes depend largely on the molecules that they release into and uptake from the extracellular space, also referred to as the microenvironment, that astrocytes and neurons share (Niranjan, 2014). Astrocytes usually play a neuroprotective role, which in some cases can lead to neurotoxicity. For example, when astrocytes undergo gliosis in response to neuronal injury or toxic insults, together with microglia, they release cytokines and chemokines that are deleterious to neurons (Mena and Garcia, 2008). An increased number of activated microglial cells has consistently been reported in PD (Tambuyzer et al., 2008). Microglial cells represent resident brain macrophage cells and can be transformed into activated cells. Activated microglial cells may have a deleterious effect on dopaminergic neurons. In PD, there is an increased density of glial cells that release pro-inflammatory and potentially cytotoxic substances such as cytokines TNF-α and IL-6 (Rogers et al., 2007). It has been reported that modified, aggregated, or overexpressed α-syn can play an important role in glial cell activation (Zhang et al., 2005; Béraud et al., 2013). Aggregated α-syn stimulates activated microglial cells to produce pro-inflammatory cytokines such as IL-6, IL-1, and TNF-α, which may be detrimental for dopaminergic neurons (Bessler et al., 1999).

In this article, we demonstrated that injection of α-syn-98 aggregates results in significant increases in GFAP and Iba1 protein levels at 4 weeks after injection into the SN. This indicates that injecting α-syn-98 aggregates into the SN induces robust glial cell activation. This observation was confirmed by IHC and western blot analysis. Taken together, these results suggest glial cell involvement in the pathological process of dopaminergic neuronal damage. It is believed that in PD, overexpression of α-syn stimulates microglia, which results in the release of pro-inflammatory cytokines and reactive oxygen species (ROS) that accelerate inflammation and neurodegeneration (Halliday and Stevens, 2011). It is well established that misfolded α-syn directly activates microglia, inducing production and release of pro-inflammatory cytokines such as IL-6 and TNF-α (Béraud et al., 2013). There is evidence indicating that cytokines, including IL-6 and TNF-α, are significantly elevated in PD (McCoy et al., 2011). In our study, we observed an increase in IL-6 and TNF-α levels at 4 weeks after α-syn-98 aggregates injection into the SN. Increases in levels of NF-κB/p65 the midbrain was also observed after injection of α-syn-98 aggregates. These results suggest that the NF-κB pathway and inflammatory cytokines may play an important role in the degeneration of dopaminergic neurons.

Previous studies have shown that overexpression of human α-syn results in NF-κB activation, localized inflammation, microglial activation, and gradual degeneration of dopaminergic neurons (Theodore et al., 2008). In the human PD brain, there is evidence for increased expression and nuclear translocation of NF-κB/p65 protein, and similar results were observed in MPTP-intoxicated mice (Ghosh et al., 2007). NF-κB is an inducible nuclear transcription factor that regulates expression of many genes and may function as a master switch in a variety of immune and inflammatory processes (Tsoulfas and Geller, 2001). Promoter regions of many pro-inflammatory cytokines contain DNA binding sites for NF-κB family proteins (Hayden and Ghosh, 2004). We found that NF-κB/p65 and pro-inflammatory cytokines were elevated in the midbrain after injection α-syn-98 aggregates. Thus, α-syn-98 aggregates may act through the NF-κB pathway to generate an inflammatory response that leads to dopaminergic neuron cell death and neurodegeneration.

In summary, our results have shown that injection of recombinant human α-syn-98 aggregates into the SN induces a neurodegenerative response in mice, which is characterized by an accumulation of endogenous α-syn and degeneration of nigrostriatal dopaminergic neurons. Our results suggest that α-syn-98 aggregates may lead to the activation of glial cells and initiation of the NF-κB pathway. Activation of NF-κB, which is a key transcription factor, can result in the release of a variety of neurotoxic substances and promote oxidation reactions that injure neurons in the SN. Our study provides an evidence for the involvement of α-syn-98 in the pathogenesis of PD. In addition, it provides a progressive and neurodegenerative model of synucleinopathy in the mouse and helps us further understand the pathogenesis of α-syn in PD. However, further studies are required to elucidate the mechanism of α-syn-98 in PD.

## Data Availability

The raw data supporting the conclusions of this manuscript will be made available by the authors, without undue reservation, to any qualified researcher.

## Ethics Statement

This study was carried out in accordance with the recommendations of the Yunnan Province Experimental Animal Management Association and the Experimental Animal Ethic Committee of the Institute of Medical Biology Chinese Academy of Medical Sciences. The protocol was approved by the Experimental Animal Ethic Committee of the Institute of Medical Biology Chinese Academy of Medical Sciences.

## Author Contributions

KM and TD conceived the idea and designed the experiment. TD and ZW performed the main experiments, analyzed the data and co-wrote the main manuscript. HL participated in this work. KM and SL revised the manuscript. All authors reviewed the manuscript.

## Conflict of Interest Statement

The authors declare that the research was conducted in the absence of any commercial or financial relationships that could be construed as a potential conflict of interest.

## Footnotes

^1^http://atlas.brain-map.org/
